# GalNAc glycoprotein expression by breast cell lines, primary breast cancer and normal breast epithelial membrane

**DOI:** 10.1054/bjoc.2001.2028

**Published:** 2001-09

**Authors:** S A Brooks, D M S Hall, I Buley

**Affiliations:** School of Biological & Molecular Sciences, Oxford Brookes University, Gipsy Lane, Headington, Oxford, OX3 0BP, UK; Department of Cellular Pathology, John Radcliffe Hospital, Headington, Oxford, OX3 9DU, UK

**Keywords:** *Helix pomatia* lectin, HPA, GalNAc glycoproteins, breast cancer, metastasis, cell lines

## Abstract

Over-expression of N-acetylgalactosamine glycoproteins as detected by binding of the lectin from *Helix pomatia* (HPA), is associated with metastatic competence and poor patient prognosis in a range of human adenocarcinomas. These glycoproteins remain poorly characterised, and their functional role has yet to be elucidated. This study describes characterisation of a range of human breast/breast cancer cell lines for the expression of the N-acetylgalactosaminylated glycoproteins of interest, and their comparison with normal breast epithelium and a range of clinical breast carcinoma samples. Confocal and light microscopy studies revealed cytochemical HPA-binding patterns consistent with a fundamental disruption in normal glycobiosynthetic pathways attending increasing metastatic potential. We report the most complete comparative analysis of HPA-binding ligands from cultured breast cells, clinical breast carcinoma samples and normal breast epithelium to date. Lectin blotting identified 11 major HPA-binding glycoprotein bands common to both clinical tumour samples and breast cell lines and 6 of these bands were also expressed by samples of normal breast epithelium, albeit at much lower levels. Moreover, very marked quantitative but not qualitative differences in levels of expression consistent with metastatic capability were noted. © 2001 Cancer Research Campaignhttp://www.bjcancer.com


*British Journal of Cancer* (2001) **85**(7), 1014–1022
© 2001 Cancer Research Campaign
doi: 10.1054/ bjoc.2001.2028, available online at http://www.idealibrary.com on IDEAL

http://www.bjcancer.com

### GalNAc glycoprotein expression by breast cell lines, primary breast cancer and normal breast epithelial membrane

**SA Brooks[1], DMS Hall[1] and I Buley[2]**

[1]School of Biological & Molecular Sciences, Oxford Brookes University, Gipsy Lane, Headington, Oxford, OX3 0BP, UK; [2]Department of Cellular Pathology, John Radcliffe Hospital, Headington, Oxford, OX3, 9DU, UK

**Summary** Over-expression of N-acetylgalactosamine glycoproteins as detected by binding of the lectin from *Helix pomatia* (HPA), is associated with metastatic competence and poor patient prognosis in a range of human adenocarcinomas. These glycoproteins remain poorly characterised, and their functional role has yet to be elucidated. This study describes characterisation of a range of human breast/breast cancer cell lines for the expression of the N-acetylgalactosaminylated glycoproteins of interest, and their comparison with normal breast epithelium and a range of clinical breast carcinoma samples. Confocal and light microscopy studies revealed cytochemical HPA-binding patterns consistent with a fundamental disruption in normal glycobiosynthetic pathways attending increasing metastatic potential. We report the most complete comparative analysis of HPA-binding ligands from cultured breast cells, clinical breast carcinoma samples and normal breast epithelium to date. Lectin blotting identified 11 major HPA-binding glycoprotein bands common to both clinical tumour samples and breast cell lines and 6 of these bands were also expressed by samples of normal breast epithelium, albeit at much lower levels. Moreover, very marked quantitative but not qualitative differences in levels of expression consistent with metastatic capability were noted. © 2001 Cancer Research Campaign http://www.bjcancer.com

**Keywords**: *Helix pomatia* lectin; HPA; GalNAc glycoproteins; breast cancer; metastasis; cell lines

There is increasing evidence that changes in cellular glycosylation attend alterations in cell behaviour in both normal and in pathological processes, and that this may be of particular interest in malignancy. The lectin from *Helix pomatia* (HPA) has emerged as an interesting marker of altered glycosylation in cancer, where expression of glycoconjugates recognised by the lectin appear to be associated with poor patient prognosis (see Mitchell and Schumacher, 1999; Brooks, 2000 for review). This association has been reported in breast cancer (Fenlon et al, 1987; Leathem and Brooks, 1987; Fukutomi et al, 1989, 1991; Alam et al, 1990; Noguchi et al, 1993a, 1993b, 1994; Thomas et al, 1993), and also in adenocarcinomas at other common sites, including gastric cancer (Macartney, 1986; Kakeji et al, 1991; Maehara et al, 1995; Okuyama et al, 1998), carcinoma of the oesophagus (Yoshida et al, 1993, 1994a,b) colorectal carcinoma (Ikeda et al, 1994; Schumacher MD et al, 1994a, 1994b), carcinoma of the thyroid (Sasano et al, 1989) and prostate cancer (Shiraishi et al, 1992). Only one published study has failed to confirm this relationship (Gusterson et al, 1993), and it appears that this may have been the fault of the inappropriate methodology employed (Brooks et al, 1996).

As a prognostic marker, HPA binding has been shown to be independent of other established prognostic factors (including tumour size, histological grade, tumour type, S-phase fraction, ploidy) except for nodal status, a very physical indication that the tumour is metastatically competent, and with which HPA binding is very closely associated (Brooks et al, 1993). In view of the increasing evidence of the roles of oligosaccharides in a whole variety of cell communication and cell adhesion events, research into the potential involvement of HPA-binding glycoforms in the biology of the metastatic cascade is warranted. Currently, this is an area that has been under-researched.

HPA has a limited binding repertoire, in that, unusually for a lectin, its combining site may be no larger than a single terminal non-reducing α-N-acetylgalactosamine residue (Hammarstrom and Kabat, 1969), and it will thus recognise any glycoconjugate terminating in this structure. Preliminary analyses have indicated that HPA recognises a heterogeneous range of glycoconjugates in breast cancer, including, amongst others, glycoproteins bearing the Tn epitope (N-acetylgalactosamine-O-Ser/Thr) and blood group A substance (Brooks et al, 1993). However, the HPA-binding partners have been imprecisely characterised, and it remains unclear whether different tumour samples express the same, or different, profiles of HPA-binding molecules, and whether any, or all of these are expressed by 'normal' cells. Perhaps more crucially, there has been little or no research into their potential involvement in metastatic mechanisms.

The study described in this paper therefore has two principal aims. Firstly, as a prelude to investigating the role(s) of HPA-binding glycoconjugates in the processes of breast cancer metastasis, we wished to characterise a range of breast cell lines (derived from either 'normal' breast epithelium, primary breast carcinoma or breast carcinoma of metastatic origin) for their expression of HPA-binding glycoconjugates in order to establish their suitability

*Received 5 December 2000*
*Revised 18 June 2001*
*Accepted 2 July 2001*

for the development of in vitro models of metastasis. This has been approached by comparison of HPA-binding characteristics at the cytochemical level and subsequent analysis of HPA-binding glycoproteins with those of human clinical tumour samples and normal breast epithelium. Secondly, and as a result of this analysis, we wished to clarify whether different tumour and cell line samples produce the same or different profiles of HPA-binding glycoproteins, and whether these were unique to malignant cells, or also produced by normal breast epithelial cells.

### MATERIALS AND METHODS

### Cell lines

7 breast epithelial cell lines were chosen to provide a range of phenotypic and behavioural characteristics ranging from ‘normal’ to highly tumorigenic/metastatic. Details are listed in Table 1. Cells were cultured in Dulbecco’s Modified Eagles Medium F-12 (DMEM F-12, Gibco-BRL) supplemented with 10% heat inactivated fetal calf serum (FCS) (Harlan Sera-Labs), 2 mM L-glutamine (Gibco-BRL) and 50 μg $ml^{-1}$ penicillin and streptomycin (Gibco-BRL). Adherent cell monolayers were routinely harvested for passaging using 0.05% trypsin-0.02% EDTA (Gibco-BRL).

### Preparation of cultured cells for light microscopy

Cells were cultured for 24 hours in the absence of FCS, monolayers were scraped from the culture flask and washed in Tris-buffered saline (TBS), pH 7.6, containing 0.02% w/v $MgCl_2$, 0.01% w/v $CaCl_2$. As previous clinical studies on HPA binding have employed routinely fixed and processed paraffin wax-embedded tissue, cells were treated in a comparable manner. They were fixed overnight at 4˚C in 4% v/v formol saline, washed in TBS and resuspended in a drop of 4% w/v low melting point agarose (Sigma). Cell/agarose pellets were then processed to paraffin wax by conventional protocol as employed to routinely process surgical specimens.

### Preparation of cultured cells for confocal microscopy

Cells were grown on sterile (size 0) glass coverslips for 24 hours in the absence of FCS to near confluence. Cells were washed briefly in TBS, permeabilised with 0.1% v/v Triton X-100 for 10 minutes, washed, and blocked in 2.5% w/v bovine serum albumin (BSA) in TBS.

### Preparation of cells for flow cytometry

Cells were incubated overnight in the absence of FCS and routinely harvested. Cells were resuspended to $10^6$ cells in washing medium (TBS with 1% w/v sodium azide and 2% v/v FBS).

### Preparation of cell lysates for SDS-PAGE and HPA lectin blotting

Cells were lysed in NP40 lysis buffer (1% w/v NP40, 0.5% w/v sodium deoxycholate, 0.05% SDS) containing protease inhibitors, for 30 minutes, on ice.

### Clinical tumour samples

19 breast carcinomas excised at The John Radcliffe Hospital, Oxford were included in this study. 15 were infiltrating ductal carcinomas and 4 were infiltrating lobular carcinomas. Both fresh and routinely fixed and processed, paraffin wax-embedded samples were available for all cases.

### Preparation of fresh tumour samples for SDS-PAGE and blotting

Tumour tissue was dissected from surrounding fat and macroscopically normal breast tissue. Samples were homogenised in ice cold homogenisation buffer with protease inhibitors (400 mM KCl, 2 mM EDTA, 20 mM MOPS, 10 mM MgCl, 10% v/v glycerol in distilled water), then filtered through a 0.2 μm filter (Acrodisc) and dialysed overnight at 4˚C against several changes of TBS.

### Normal breast epithelial membrane – milk fat globule membrane (MFGM)

12 samples of MFGM from healthy, lactating female donors were included in this study.

### HPA cytochemistry at the light microscope level

5 μm thick paraffin wax-embedded sections of clinical tumour samples and cultured cells were stained for the binding of HPA by

**Table 1** Characteristics of breast and breast cancer cell lines employed in this study

| Cell line | Originator | Reference |
|---|---|---|
| HMT 3522 | Established from benign fibrocystic breast tissue. Represents normal breast epithelium. Spontaneously immortalised, non-transformed. Non-tumorigenic in nude mice. Cells retain polarity in culture. | Briand et al, 1987 |
| BT 474 | Derived from primary breast cancer. Do not readily produce tumours in nude mice. Cells retain polarity in culture. | Lasfargues et al, 1978 |
| MDA MB 435 | Derived from a pleural effusion of infiltrating ductal cancer. Parent strain poorly tumorigenic in nude mice. | Cailleau et al, 1974 |
| MDA MB 468 | Derived from a pleural effusion of infiltrating ductal cancer. Poorly tumorigenic in nude mice. | Filmus et al, 1985 |
| ZR 751 | Derived from malignant ascites from primary infiltrating ductal cancer. Tumorigenic in nude mice in the presence of oestrogen pellets. | Engel et al, 1978 |
| MCF 7 | Derived from malignant pleural effusion from primary infiltrating ductal cancer. This strain is tumorigenic in nude mice. | Soule et al, 1973 |
| DU 4475 | Derived from cutaneous metastatic nodule of primary breast origin. Tumorigenic in nude mice. Poorly differentiated. | Langlois et al, 1979 |

*© 2001 Cancer Research Campaign*

the indirect method described previously by Brooks and Leathem (1991). Specificity of binding was confirmed by incubating sections with lectin in the presence of 0.1 M GalNAc (Sigma). For negative control, lectin was omitted. For positive control, a case previously known to be strongly positive for HPA binding was included.

### HPA cytochemistry for confocal microscopy

Cells were incubated with 5 µg ml$^{-1}$ HPA-FITC conjugate (Sigma) (with 2.5% BSA in TBS) in the dark, on ice, for 30 minutes. After washing thoroughly in TBS, cells were fixed in a 4% (v/v) formaldehyde solution for 15 minutes, washed, and nuclei labelled by brief incubation with 3 µg ml$^{-1}$ propidium iodide (Sigma). They were then mounted in citifluour mountant (Agar Scientific). Confirmation of specificity of labelling and negative controls were as described previously.

### HPA cytochemistry for flow cytometry

Cells were incubated in blocking medium (TBS with 0.25% w/v BSA) for 15 minutes at room temperature. $10^6$ cells were suspended in 3 µg ml$^{-1}$ HPA-FITC (Sigma) conjugate in the dark for 30 minutes, on ice, and then washed in several changes of TBS before fixation in 4% v/v formol saline for 2 hours at 4˚C. Unlabelled cells were included as a control to assess levels of auto-fluorescence and confirmation of specificity of labelling was performed as described previously.

### Flow cytometry

All analyses were carried out on a FACScan (Becton Dickinson).

### SDS-PAGE and lectin blotting

Samples were subjected to chloroform/methanol precipitation according to the method described by Wessel and Flugge (1984). They were dissolved 1:1 in sample buffer, pH 6.8 (0.0625M Tris, 4% w/v SDS, 5% w/v dithiothreitol, 18% w/v glycerol, 0.025% w/v bromophenol blue) and placed in a boiling water bath for 2 minutes. 20 µg of total protein for each of the samples was loaded per lane. Electrophoresis was performed under denaturing conditions using the discontinuous buffer system described by Laemmli (1970). Separated glycoproteins were then transferred to pure nitrocellulose membrane (Schliecher and Schuell) using a 'Fastblot' semi-dry electroblotting system (Biometra) using the protocol described by Towbin et al (1979).

### Probing blots for HPA binding

All dilutions, incubations and washes were performed using TBS with 0.1% v/v Tween-20 (TBST) and were carried out on a rocking platform at room temperature unless otherwise stated. Blots were blocked in 2.5% w/v BSA (Sigma) for 30 minutes. They were incubated in 1 µg ml$^{-1}$ biotinylated HPA (Sigma) in TBS-1% w/v BSA overnight, then with 2 µg ml$^{-1}$ horseradish peroxidase conjugated avidin (Sigma) for 1.5 hours and developed using the enhanced chemiluminescence (ECL) system (Amersham Life Sciences), according to the manufacturer's instructions. Confirmation of specificity of labelling and negative controls were as described previously.

### RESULTS

### HPA cytochemistry at the light microscope level

*Cell lines*

The cell lines displayed marked differences in HPA-binding characteristics as illustrated in Figure 1 and summarised in Table 2. HMT 3522, derived from 'normal' breast epithelial cells, exhibited negligible levels of HPA binding whereas all cell lines derived from malignant breast epithelium showed some detectable HPA binding. Significantly, levels of expression correlated well with known tumorigenic and metastatic capability of the cells, with more tumorigenic/metastatic lines exhibiting higher levels of expression of HPA-binding glycoconjugates than those derived from primary breast cancer (BT 474) or non-malignant breast epithelium (HMT 3522). All cell lines derived from metastatic cancer cells exhibited marked cell surface as well as significant cytoplasmic labelling. Omission of the lectin or incubation in the presence of 0.1 M GalNAc completely abolished labelling.

*Clinical tumour samples*

Tumour samples showed variable levels of HPA binding. One case was judged HPA negative (5%+/–). All others were judged HPA positive, and staining percentage and intensity ranged from 50%

**Table 2** Full summary of the HPA-binding characteristics of the cell lines using HPA cytochemistry (both at the light and the confocal microscope level) and flow cytometry approaches. Levels of HPA labelling is indicated by the number of + designated, where – denotes no labelling, +/– denotes negligible labelling and ++++ denotes intense labelling

| Cell line | HPA cytochemistry on cultured breast cell lines | | | Flow cytometry (cell surface labelling) | |
|---|---|---|---|---|---|
| | Cell line sections (for light microscopy) | Whole cells (confocal microscopy) | | | |
| | | Perinuclear labelling | Cell surface/cytoplasmic labelling | % cells +ve | Relative intensity |
| HMT 3522 | – | – | +/– | 3 | 3.9 |
| BT 474 | + | – | + | 31 | 10.4 |
| MDA MB 435 | + | +/– | +/– | 4.89 | 9.5 |
| MDA MB 468 | ++ | ++ | +/– | 19.42 | 19.7 |
| ZR 75 1 | +++ | +++ | ++ | 73.56 | 51.9 |
| MCF 7 | ++++ | ++++ | +++ | 98.66 | 65.9 |
| DU 4475 | ++++ | ++++ | +++ | 98.34 | 107.1 |

 *© 2001 Cancer Research Campaign*

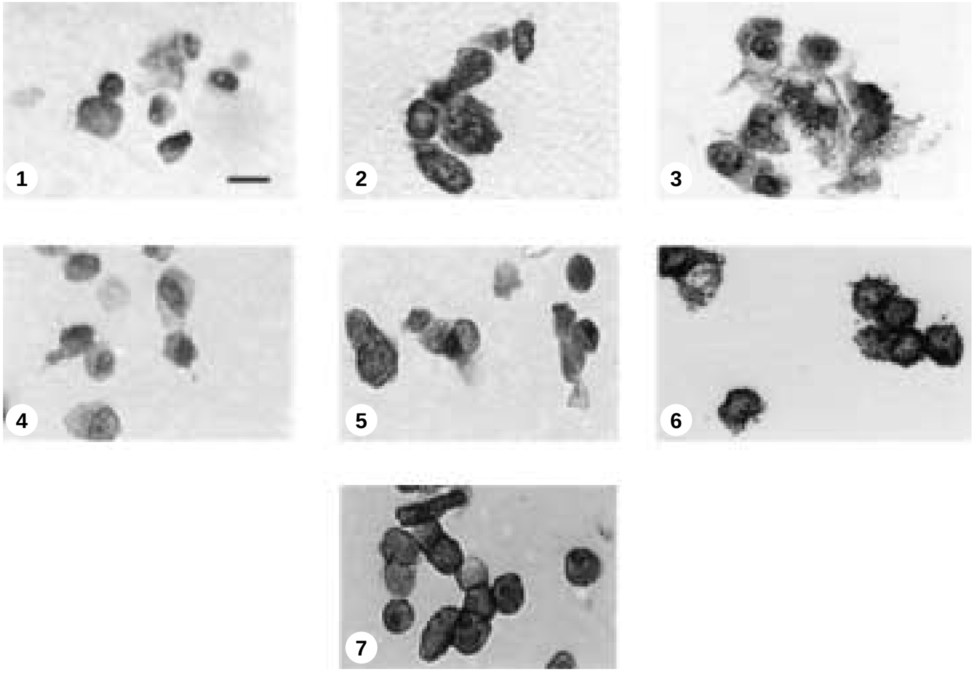

**Figure 1** HPA immunoperoxidase labelling of cell lines analysed at the light microscope level. Counterstain haematoxylin. Panels 1–7 as follows: **(1)** HMT 3522; **(2)** BT 474; **(3)** MDA MB 435; **(4)** MDA MB 468; **(5)** ZR 75 1; **(6)** MCF 7; **(7)** DU 4475. Levels of labelling ranged from negligible (HMT 3522), weak (BT 474) to intense (DU 4475). Size bar = 15 μm

++ to 100% ++++. In some cases, distinct morphologically identical sub-populations of cells negative and strongly positive for HPA binding were apparent within the same tumour mass. Representative examples of staining in 4 cases are illustrated in Figure 2. Omission of the lectin or incubation in the presence of 0.1 M GalNAc completely abolished labelling.

Clinical follow up was possible in 18 of the 19 cases. The single HPA-negative patient remained alive and well with no sign of recurrence. Of the 17 HPA-positive cases for whom follow up was available, 9 remained alive and well with no sign of recurrence, 3 had suffered recurrences but were alive, and 5 were dead. Specific details relating to the clinical follow up of the 4 cases illustrated in Figure 2 is given in Table 3.

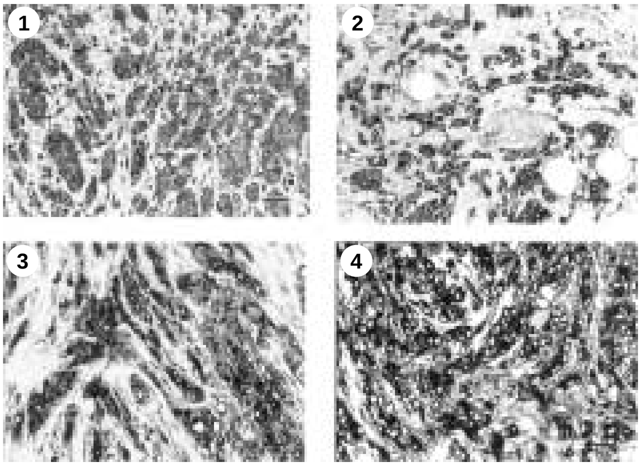

**Figure 2** HPA immunoperoxidase labelling of clinical tumour samples. Counterstain haematoxylin. Panels 1–4 illustrate representative breast cancer samples displaying a range of HPA labelling as follows: **(1)** 5% (+/–); **(2)** 40% (++); **(3)** 80% (+++); **(4)** 100% (++++). These are the same cases as those analysed by HPA lectin blotting illustrated in Figure 4C. Size bar = 25 μm

### HPA cytochemistry of cell lines at the confocal microscope level

Very low levels of punctate, diffuse cytoplasmic HPA labelling were detectable in HMT 3522 cells. BT 474 cells exhibited some cell surface localisation, but no intracytoplasmic labelling. All cell lines derived from metastatic breast cancer exhibited marked and striking intracellular localisation of HPA labelling in addition to varying levels of cell surface labelling. Intense perinuclear localisation of HPA labelling, possibly representative of Golgi apparatus or endoplasmic reticulum labelling was observed, and was particularly marked in DU 4475 and MCF 7 cells. Results are illustrated in Figure 3 and summarised in Table 2. Omission of the lectin or incubation in the presence of 0.1 M GalNAc completely abolished labelling.

### Analysis of cell surface HPA labelling of the cell lines by flow cytometry

Levels of cell surface labelling were consistent with the results of HPA cytochemistry at light and confocal microscope levels, as summarised in Table 2. Omission of the lectin showed very low levels of autofluorescence and incubation in the presence of 0.1 M GalNAc completely abolished labelling.

### SDS-PAGE and HPA lectin blotting

### *Milk fat globule membrane samples*

6 HPA-binding bands were detectable in all samples; representative results are illustrated in Figure 4A. These bands were also present in the tumour and cell line samples as is clear from

© 2001 Cancer Research Campaign

**Table 3** HPA binding characteristics and clinical data relating to cases illustrated in Figures 2 and 4(C)

| Case no. | Tumour type | Grade | Nodal status | HPA binding | Follow up at 5 years |
|---|---|---|---|---|---|
| 1 | Infiltrating tubular | I | Positive | 5%+ /– (negative) | Recurrence free, alive and well |
| 2 | Infiltrating ductal | I | Positive | 65% ++ | Recurrence 1400 days post operatively. Alive |
| 3 | Infiltrating ductal | I | Positive | 80% +++ | Died 493 days post operatively |
| 4 | Infiltrating ductal | II | Unknown | 100% ++++ | Died 1503 days post operatively |

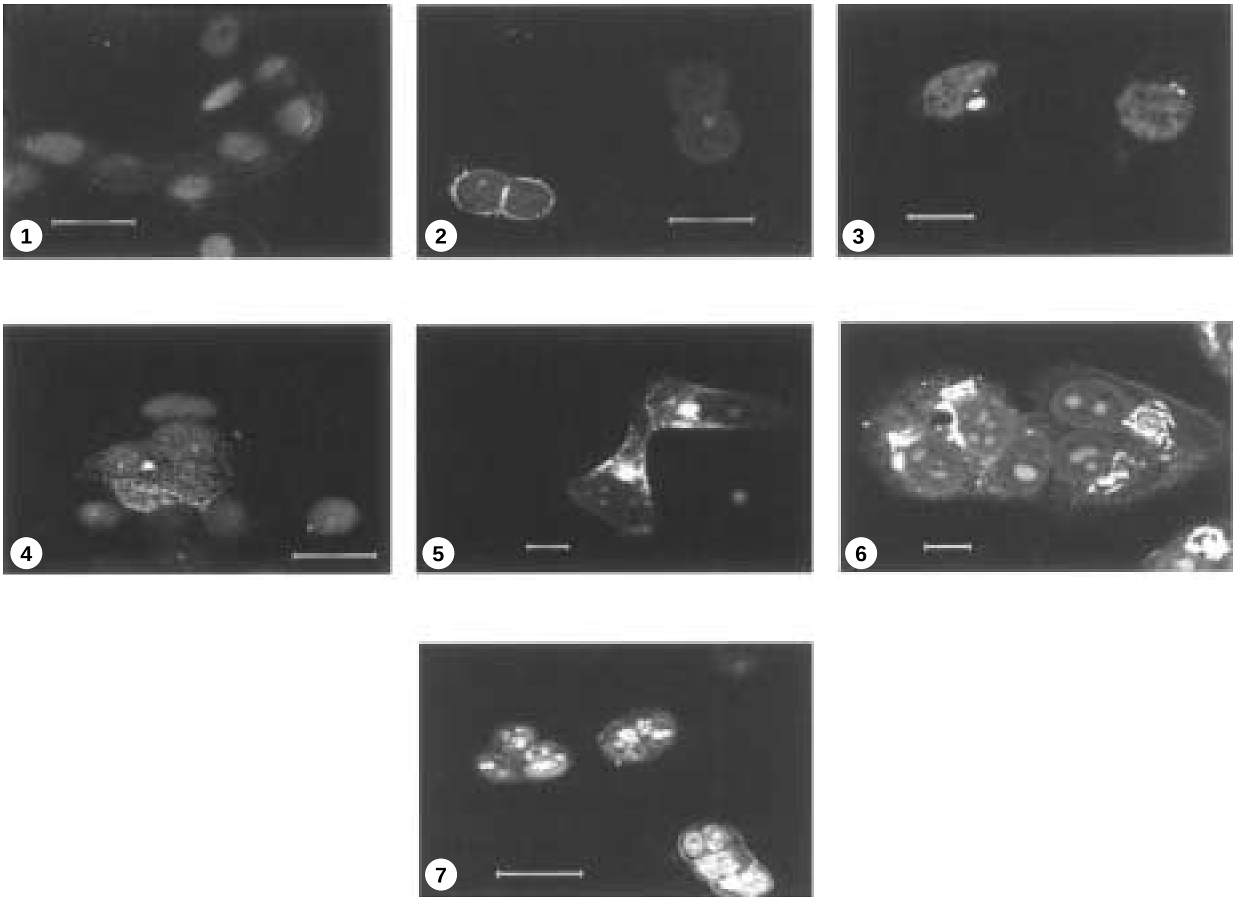

**Figure 3** FITC-HPA labelling of cell lines, counterlabelled with the nuclear label propidium iodide, analysed by confocal microscopy. Panels 1–7 as follows: **(1)** HMT 3522; **(2)** BT 474; **(3)** MDA MB 435; **(4)** MDA MB 468; **(5)** ZR 75 1; **(6)** MCF 7; **(7)** DU 4475. HPA labelling intensity is consistent with that observed at the light microscope level. Localisation of labelling is as follows: HMT 3522 (panel 1) negligible levels of punctate cytoplasmic labelling; BT 474 (panel 2) low levels of cell surface labelling only; all of the other metastatically derived cells (panels 3–7) show marked perinuclear labelling as well as varying levels of cell surface labelling. Size bar = 15 μm comparison of Figures 4A, B and C. Levels of expression were very low, and bands were only detectable after extended blot development (24 hours).

### *Cell lines*

An identical range of 11 major HPA-binding glycoproteins was present in the lysates of all 7 cell lines analysed. There were marked quantitative differences in levels of expression, with HMT 3522 expressing the lowest levels, BT 474 expressing slightly higher levels, and all the cell lines derived from metastatic breast cancer expressing significantly higher levels. This is readily observed in Figure 4B.

The 6 bands detected in the MFGM blots were also identified in the cell line blots.

### *Breast tumour samples*

Satisfactory analysis of lysates was achieved for 16 of the 20 breast cancer samples. There was no *qualitative* difference in expression of HPA binding between samples, but highly marked *quantitative* differences in the levels of expression were seen. Representative results are illustrated in Figure 4C. The samples are the same as those illustrated in Figure 2. The sample run in track 1 was 5% (+/–) for HPA binding by lectin histochemistry, track 2 was 65% (++), track 3 was 80% (+++) and track 4 was 100% (++++). Intensity of bands detectable on the blot corresponds well to the lectin histochemistry results on the same samples. Again, clinical details of these cases are summarised in Table 3.

The 11 major bands detected in the cell line blots were also identified in the blots of glycoproteins derived from the clinical samples.

 *© 2001 Cancer Research Campaign*

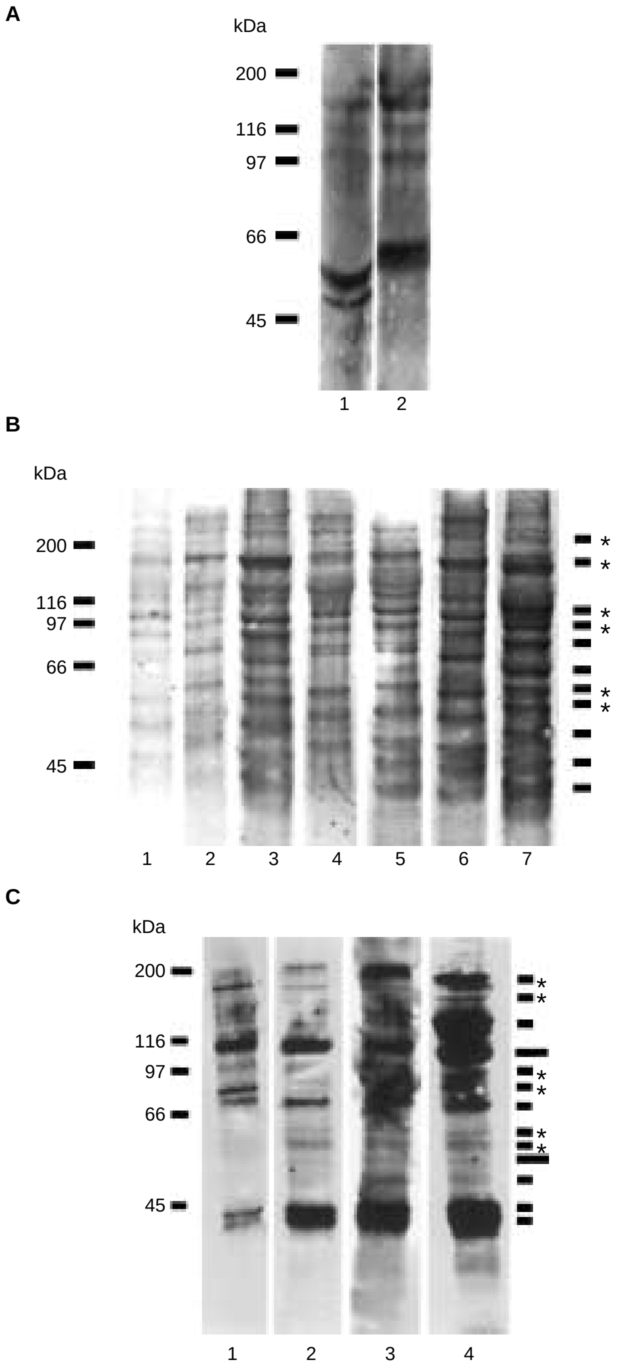

**Figure 4** HPA lectin blots. All blots were probed with HPA. MW markers (kDa) are indicated to the left of the lanes. 20 μg total protein loaded per lane. **(A)** MFGM samples. Identical profiles were observed in all samples analysed and 2 representative tracks are illustrated here. In all samples analysed, 6 weak HPA binding bands were detectable after extended development (yielding high background). **(B)** Cell line lysates. Lanes 1–7 as follows: **(1)** HMT 3522; **(2)** BT 474; **(3)** MDA MB 435; **(4)** MDA MB 468; **(5)** ZR 75 1; **(6)** MCF 7; **(7)** DU 4475. Levels of expression of the HPA-binding bands are quantitatively different with HMT 3522 showing the lowest levels of expression and DU 4475 showing the highest. Qualitatively the banding pattern is comparable, with 11 shared major HPA binding bands * apparent. Of these, 6 appeared to correspond to those detectable in the MFGM samples **. **(C)** Clinical tumour samples. Samples are the same as in Figure 2. The levels of expression of the HPA-binding bands are quantitively different with sample 1 showing the lowest levels and sample 4 the highest, consistent with the cytochemistry results. The 11 bands detectable in the cell line samples* were also apparent in all the tumour samples, and extra bands*** were also detected. ▬ marked; ▬* marked; ▬▬ marked

### DISCUSSION

The cell lines employed in this study were chosen to represent a range of breast epithelial sources ranging from 'normal' to malignant and highly tumorigenic/metastatic. HPA cytochemistry at the light microscope level revealed that the cells exhibited variable degrees of HPA binding ranging from virtually undetectable in cells derived from 'normal' breast epithelium (HMT 3522), weak in cells derived from primary breast cancer (BT 474), to very intense levels of expression in cells derived from metastatic breast cancers. A range of HPA-binding patterns were also observed in the clinical tumour samples analysed in this study, ranging from negative to intense binding, with some samples exhibiting subpopulations of cancer cells positive and negative for HPA binding within a single tumour sample. This has also been a frequent observation in other studies (for example, Leathem and Brooks, 1987; Brooks and Leathem, 1991). Cellular staining patterns in clinical samples and cell lines were similar. DU 4475, for example, exhibited intense cell border localisation of HPA labelling; whereas in others, for example, MDA MB 435, localisation appeared at the light microscope level to be more diffusely cytoplasmic. These patterns have been reported in studies on clinical samples (for example, Leathem and Brooks, 1987; Brooks and Leathem, 1991). Flow cytometry confirmed levels of cell surface labelling with HPA consistent with light microscopy results.

Confocal laser scanning microscopy facilitated more detailed analysis of the distribution of HPA-binding glycoconjugates within the cultured cells. Negligible labelling of 'normal' HMT 3522 cells was noted. BT 474, derived from primary breast cancer, exhibited cell surface expression, with no evidence of polarity in expression or any cytoplasmic labelling. All cell lines derived from metastatic breast cancer exhibited, in addition to cell-surface binding, striking localisation in a discrete area in the perinuclear region of the cytoplasm, possibly corresponding to Golgi apparatus or endoplasmic reticulum. These observations may be consistent with increasing disruption in glycosylation pathways in the Golgi apparatus/endoplasmic reticulum, or in transport pathways, leading to a concentration of immature N-acetyl-galactosaminylated glycoforms in these cellular compartments. Previous ultrastructural (electron microscopy) studies of cultured breast cell lines have failed to report HPA labelling of the Golgi apparatus (Calafat and Jannssen, 1984; Mitchell et al, 1995), but studies of non-malignant cells from sources other than breast have reported HPA localisation to this organelle, consistent with its role in glycosylation (Roth, 1984; Laitinen et al, 1990; Virtanen, 1990).

Analysis of HPA-binding glycoproteins by SDS-PAGE and HPA lectin blotting revealed that the cell lines all expressed apparently identical profiles in terms of the 11 major glycoprotein bands detected. Intriguingly, there was a very marked *quantitative* difference in levels of expression, which was readily detectable by eye. HMT 3522, derived from 'normal' breast epithelium, expressed the lowest levels; BT 474, derived from primary breast cancer, expressed moderately low levels; whereas the cell lines derived from metastatic breast cancer all expressed quantitatively greater levels of HPA-binding glycoproteins, entirely consistent with the results obtained by HPA cytochemistry at both the light and confocal level and also by flow cytometry.

In all cell lines, 11 major glycoprotein bands were detected by HPA lectin blotting. These bands ranged in molecular weight from 20 to 200 kDa. Previously, Schumacher et al (1995) and Mitchell et al (1995) reported between 4 and 7 HPA-binding bands ranging

*© 2001 Cancer Research Campaign* 

in molecular weight from 20–90 kDa. Including a band at 69 kDa, which the authors tentatively identified as an N-acetylgalactosaminylated form of albumin, and a band at 90 kDa, tentatively identified as the transferrin receptor.

The analysis of lysates of primary breast cancers reported here revealed a complex and heterogeneous array of 13 major HPA-binding bands. Levels of expression differed markedly between tumours, but *qualitatively* the profile of HPA-binding moieties appeared entirely consistent between samples, and the 11 major bands detected on the cell line blots were readily apparent on the blots of the clinical samples. As in the cell line analysis, we report a more complex profile to that reported in earlier studies, and report expression of a similar range of HPA-binding species in different samples. Previous analyses of primary breast cancer, and metastases to lung, bone and liver have revealed an array of less than 10 HPA-binding bands, quantitatively different between samples, but as in the study reported here, with marked qualitative similarities in the bands expressed (Brooks and Leathem, 1995; Streets et al, 1996). The methodology employed in the current study, using enhanced chemiluminescence (ECL), facilitates a much greater level of sensitivity than that obtained by the peroxidase/diaminobenzidine method employed in earlier studies by ourselves (Brooks and Leathem, 1995; Streets et al, 1996) and others (Mitchell et al, 1995) and this may account for the greater number of HPA-binding glycoprotein moieties detected in this study in analyses of both cell lines and clinical samples.

Clearly, in a study of only 20 cases with a maximum of 5 years follow up, only limited conclusions can be drawn regarding the clinical significance of quantitative differences in expression of HPA binding glycoproteins. However, it is noteworthy that the single case judged to be HPA negative – panel 1 in Figure 2 and track 1 in Figure 4(C) – remained alive and well with no sign of recurrence, in spite of being node positive at the time of diagnosis, while the 3 HPA-positive cases for which detailed analysis is presented – panels 2 (65% ++), 3 (80% +++) and 4 (100% ++++) in Figure 2 and tracks 2, 3 and 4 respectively in Figure 4(C) – had suffered recurrences and/or died. Of the remaining 14 HPA-positive cases in the study for whom we had follow up information, 5 had suffered recurrences and/or died while 9 remained alive and well at 5 years. We have previously determined (unpublished observations) that there is no prognostic significance associated with increasing levels of HPA positivity of cases as determined by lectin histochemistry. Tumours tend to be either completely or almost completely negative for HPA binding, which is indicative of favourable prognosis, or, exhibit substantial sub-populations of cells positive for HPA binding, which is indicative of poor prognosis. This is true of the 20 cases examined in this study. We might speculate that if the HPA-binding glycoproteins play a functional role in metastasis, their expression by a significant sub-population of cancer cells is sufficient for dissemination to occur successfully, and expression by a larger proportion of tumour cells will have no additional clinical significance.

In the current study, we detected 6 HPA-binding glycoprotein bands in MFGM, consistent with previous reports (Streets et al, 1996). Levels of expression were extremely low in comparison to the clinical samples and cultured cells. Normal breast epithelium expresses some HPA-binding glycoproteins, as cytochemistry reveals discrete luminal surface labelling of breast ducts (Leathem and Brooks, 1987; Brooks and Leathem, 1991; Mitchell and Schumacher, 1999; Brooks, 2000). The very limited profile of HPA-binding glycoproteins, and very low levels of expression, by MFGM in comparison to breast cancers or breast cancer cell lines is consistent with a fundamental disruption in glycan processing and/or transport with transformation and tumour progression.

In previous studies, there has remained some ambiguity in interpretation of the analysis of HPA-binding glycoproteins by breast cancers and breast cell lines. A heterogeneous expression of HPA-binding glycoproteins has been apparent, but it has remained unclear whether the same, or different, HPA-binding glycoproteins are expressed by different samples. In this study, for the first time, we have addressed this issue directly. Results indicate that although both cell lines and human tumours express a highly complex array of HPA-binding moieties, and levels of expression differ quite markedly between samples, the actual profile of glycoproteins is entirely consistent. It remains to isolate and characterise these molecules further.

In view of the relationship between positive HPA labelling and metastatic competence in human breast cancer, there is clearly a need to understand the potential role(s) of these glycoconjugates in the metastatic process. From what is known about carbohydrate-receptor (lectin) interactions in many normal and disease-associated cellular processes, it appears plausible that the HPA-binding oligosaccharides might be ligands for a putative receptor (lectin) on another cell type, and thus implicated in cell–cell adhesion. An interesting paradigm is the interaction of selectins (P-selectin, L-selectin and E-selectin) with carbohydrate-binding partners during the complex mechanisms associated with leukocyte trafficking (e.g. see Zak et al, 2000), and, indeed, their possible involvement in metastasis (e.g. see Laidler and Litynska, 1997). Alternatively, the alteration in cellular glycosylation represented by the expression of HPA-binding ligands may conversely result in a failure of recognition by putative oligosaccharide receptors resulting in loss of cell–cell adhesion, which could potentially be of significance in cancer cell migration at either the primary or secondary site. It has also been suggested (Fenlon et al, 1987) that exposure of terminal GalNAc, detectable by HPA, might result from a failure of sialylation mechanisms, sialic acid being a common terminal monosaccharide which could be masking sub-terminal GalNAc in HPA-negative tumours. As sialic acid carries a negative charge, such disruption in sialylation would have profound effects on the properties of the cell surface, again affecting processes such as cell–cell adhesion. We have, however, investigated the relationship between expression of GalNAc, detectable by HPA, and expression of sialylated glycans (Brooks and Carter, 2001), and found no evidence to support a relationship between HPA positivity and failure in sialylation.

With the explosion of interest in glycobiology, and our increasing understanding of the importance of carbohydrate-receptor interactions in a whole range of biological process, including metastasis, the potential role of altered expression of N-acetylgalactosaminylated glycoproteins in breast – and other – common neoplasms warrants further study. Experiments are underway in our laboratory and early results, intriguingly, are consistent with a function in cancer cell adhesion mechanisms. Further work is required, and there remains a need to clarify the biological roles of these moieties in metastatic mechanisms, particularly in relation to cell adhesion. Only by a better understanding of the molecular complexities of the metastatic cascade can we hope to develop effective therapies to combat its effects.

The current study has demonstrated that the expression of HPA-binding glycoproteins by a range of cell lines is consistent with

 *© 2001 Cancer Research Campaign*

that observed in human tumours. Levels of expression appear to be associated with the origin of the cell types; negligible levels of expression in cells derived from 'normal' breast epithelium, low levels in cells derived from primary breast cancer, and high levels in cells derived from metastatically competent tumours. Furthermore, the cell lines display profiles of HPA-binding glycoproteins present in clinical samples. It appears, from the investigations reported here, that, taking into consideration the usual caveats regarding cell line research, that the cell lines investigated in this study may be appropriate tools for initial investigations. Moreover, this study illustrates for the first time that breast cancer cells and tissues express HPA-binding glycoproteins not detected in normal breast epithelial membranes. Furthermore, different samples and different cancer cell lines express quantitatively very different levels of HPA-binding glycoconjugates, but qualitatively very similar profiles of HPA-binding species.

### ACKNOWLEDGEMENTS

Our thanks to Professor M O'Hare at LICR/UCL Breast Cancer Laboratory for supplying the breast epithelial cell lines and Dr. A. Leathem at Department of Surgery, University College London Medical School for supplying the MFGM samples.

### REFERENCES

Alam SM, Whitford P, Cushley W, George WD and Campbell AM (1990) Flow cytometric analysis of cell surface carbohydrates in metastatic human breast cancer. *Br J Cancer* **62**: 238–242

Briand P, Petersen OW and Van Deurs B (1987) A new diploid nontumorogenic human breast epithelial cell line isolated and propagated in chemically defined medium. *In Vitro Cell Dev Biol* **23**(1): 181–188

Brooks SA (2000) The involvement of *Helix pomatia* lectin (HPA) binding N-acetylgalactosamine glycans in cancer progression. *Histology and Histopathology* **15**(1): 143–158

Brooks SA and Leathem AJC (1991) Prediction of lymph node involvement in breast cancer by detection of altered glycosylation in the primary tumour. *Lancet* **338**: 71–74

Brooks SA and Leathem AJC (1995) Expression of GalNAc glycoproteins by breast cancers. *Br J Cancer* **71**: 1033–1038

Brooks SA and Carter TM (2001) N-acetylgalactosamine, N-acetylglucosmine and sialic acid expression by primary breast cancers. *Histochem J* **103**: 37–51

Brooks SA, Leathem AJC, Campeljohn RS and Gregory W (1993) Markers of prognosis in breast cancer – the relationship between HPA binding and histological grade, SPF and ploidy. *Breast Cancer Res Treat* **25**: 247–256

Brooks SA, Lymboura M, Schumacher U and Leathem AJ (1996) Histochemistry to detect *Helix pomatia* lectin binding in breast cancer: methodology makes a difference. *J Histochem Cytochem* **44**: 519–524

Calafat J and Jannssen H (1984) Binding of lectins to human mammary tumours: ultrastructural study. *Breast Cancer Res Treat* **4**: 169–179

Cailleau R, Young R, Olive M and Reeves WJ (1974) Breast tumour cell lines from pleural effusions. *JNCI* **53**: 661–673

Engel LW, Young NA, Tralka TS, Lippman ME, O'Brien SJ and Joyce MJ (1978) Establishment and characterisation of three new continuous cell lines derived from human breast carcinomas. *Ca Res* **38**: 3352–3364

Fenlon S, Ellis IO, Bell J, Todd JH, Elston CW and Blamey RW (1987) *Helix pomatia* and *Ulex europeus* lectin binding in human breast carcinoma. *J Pathol* **152**: 169–176

Filmus J, Pollak MN, Cailleau R and Buick RN (1985) MDA-468, a human breast cancer cell line with a high number of epidermal growth factor receptors, has an amplified EGF receptor gene and is growth inhibited by EGF. *Biochem Biophys Res Comm* **128**: 898–905

Fukutomi T, Itsabashi M, Tsuane S, Yamamoto H, Nanasawa T and Hiroto T (1989) Prognostic contributions of *Helix pomatia* and carcinoembryonic antigen staining using histochemical techniques in breast carcinomas. *Jpn J Clin Oncol* **19**: 127–134

Fukutomi T, Hirohashi S, Tsuda H, Nanasawa T, Yamamoto H, Itsabashi M and Shimosato Y (1991) The prognostic value of tumour associated carbohydrate structures correlated with gene amplifications in human breast carcinomas. *Jpn J Surg* **21**: 499–507

Gusterson BA and Group T.I.L.B.C.S. (1993) Prognostic value of *Helix pomatia* in breast cancer. *Br J Cancer* **68**: 146–150

Hammarstrom S and Kabat EA (1969) Purification and characterisation of a blood group A reactive hemagglutinin from the snail *Helix pomatia* and a study of its combining site. *Biochem* **8**: 2696–2705

Ikeda Y, Mori M, Adachi Y, Matsushima T and Sugimachi K (1994) Prognostic value of the histochemical expression of *Helix pomatia* agglutinin in advanced colorectal cancer. *Dis Colon Rectum* **37**: 181–184

Kakeji Y, Tsujitani S, Mori M, Maehara Y and Sugimachi K (1991) *Helix pomatia* agglutinin binding activity is a predictor of survival time for patients with gastric carcinoma. *Cancer* **68**(11): 2438–2442

Laemmli I (1970) Cleavage of structural proteins during the assembly of the head of bacteriophage T4. *Nature (London)* **227**: 680–685

Laidler P and Litynska A (1997) Minireview: Tumor cell N-glycans in metastasis. *Acta Biochimica Polonica* **44**: 343–358

Laitinen L, Juusela H and Virtanen I (1990) Binding of the blood group reactive lectins to human adult kidney specimens. *Anat Rec* **226**: 10–17

Langlois AJ, Holder WD, Inglehart JD, Nelson-Rees WA, Wells SA and Bolognesi DP (1979) Morphological and biochemical properties of a new human breast cancer cell line. *Ca Res* **29**: 2064–2613

Lasfargues EY, Coutinho WG and Redfield ES (1978) Isolation of two human tumour epithelial cell lines from solid breast carcinomas. *JNCI* **61**: 967–978

Leathem AJ and Brooks SA (1987) Predictive value of lectin binding on breast cancer recurrence and survival. *Lancet* **i**: 1054–1056

Macartney JC (1986) Lectin histochemistry of galactose and N-acetylgalactosamine glycoconjugates in normal gastric mucosa and gastric cancer and the relationship with ABO blood group status. *J Pathol* **150**: 135–144

Maehara Y, Okuyama T, Kakeji Y, Endo K, Yamamoto M and Sugimachi K (1995) A tumour associated cell surface glycoprotein accompanying over expression and higher growth potential for gastric cancer. *Br J Cancer* **71**(5): 999–1002

Mitchell BS and Schumacher U (1999) The use of the lectin *Helix pomatia* agglutinin (HPA) as a prognostic indicator and as a tool in cancer research. *Histol Histopathol* **14**: 217–226

Mitchell BS, Vernon K and Schumacher U (1995) Ultrastructural localisation of *Helix pomatia* agglutinin – binding sites in human breast cancer cell lines and characterisation of HPA-binding glycoproteins by Western blotting. *Ultrastruc Pathol* **19**: 51–59

Noguchi M, Thomas M, Kitagawa H, Kinishita K, Kinami S, Takamura H, Miyazaki I and Mizukami Y (1993a) DNA ploidy and *Helix pomatia* lectin binding as predictors of regional lymph node metastases and prognostic factors in breast cancer. *Br Ca Res Treat* **26**: 67–75

Noguchi M, Thomas M, Kitagawa K, Ohta N, Nagamori M and Miyazaki I (1993b) Further analysis of predictive value of *Helix pomatia* lectin binding to primary breast cancer for axillary and internal mammary lymph node metastases. *Br J Cancer* **67**(6): 1368–1371

Noguchi M, Thomas M, Kitagawa H, Kinoshita K, Earashi M, Kinami SI, Takamura H, Miyazaki I and Mizukami Y (1994) *Helix pomatia* lectin and c-erbB2 expression versus axillary and internal mammary lymph node metastases in prognostic assessment of breast cancer. *Oncol Rep* **1**: 155–160

Okuyama T, Maehara Y, Kakeji Y, Tsujitani S, Korengana D and Sugimachi K (1998) Interrelation between tumour-associated cell surface glycoprotein and host immune response in gastric carcinoma patients. *Cancer* **82**: 1468–1475

Roth J (1984) Cytochemical localisation of terminal N-acetyl-D-galactosamine residues in cellular compartments of intestinal goblet cells: implications for the topology of O-glycosylation. *J Cell Biol* **98**: 399–406

Sasano H, Rojas M and Silverberg SG (1989) Analysis of lectin binding in benign and malignant thyroid tissue. *Arch Pathol Lab Med* **113**: 186–189

Schumacher U, Higgs D, Loizidou M, Pickering R, Leathem A and Taylor I (1994a) HPA binding is a useful prognostic indicator in colorectal carcinoma. *Cancer* **74**(12): 3104–3107

Schumacher U, Adam E, Flavell DJ, Boehm D, Brooks SA and Leathem AJ (1994b) Glycosylation patterns of the human colon cancer cell line HT-29 detected by *Helix pomatia* agglutinin and other lectins in culture, in primary tumours and metastases in SCID mice. *Clin Exp Metastasis* **12**(6): 398–404

Schumacher U, Adam E, Brooks SA and Leathem AJ (1995) Lectin binding properties of human cancer cell lines and human milk with particular reference to *Helix pomatia* agglutinin. *J Histochem Cytochem* **43**(3): 275–281

Shiraishi T, Atsumi S and Yatani R (1992) Comparative study of prostatic carcinoma bone metastasis amongst Japanese in Japan and Japanese, Americans and Whites in Hawaii. *Adv Exp Med Biol* **324**: 7–16

*© 2001 Cancer Research Campaign*

Soule HD, Vasquez J, Long A, Albert S and Brennan M (1973) A human breast cancer cell line from a pleural effusion derived from a breast carcinoma. *JNCI* **51**: 1409–1416

Streets AJ, Brooks SA, Dwek MW and Leathem AJ (1996) Identification, purification and analysis of a 55kDa lectin binding glycoprotein present in breast cancer tissues. *Clin Chim Acta* **254**(1): 47–61

Thomas M, Noguchi M, Fonseca L, Kitagawa H, Kinoshita K and Miyazaki I (1993) Prognostic significance of *Helix pomatia* lectin and c-erbB-2 oncoprotein in human breast cancer. *Br J Cancer* **68**(3): 621–626

Towbin H, Staehelin T and Gordon J (1979) Electrophoretic transfer of proteins from polyacrylamide gels to nitrocellulose sheets. *Proc Natl Acad Sci USA* **76**: 4350–4354

Virtanen I (1990) *Helix pomatia* agglutinin binds specifically to the Golgi apparatus in cultured human fibroblasts and reveals two Golgi apparatus specific glycoproteins. *Histochem* **94**: 397–401

Wessel D and Flugge UI (1984) A method for quantitative recovery of protein in dilute solution in the presence of detergents and lipids. *Annals Biochem* **138**: 141–143

Yoshida Y, Okamura T and Shirakusa T (1993) An immunohistochemical study of *Helix pomatia* agglutinin binding on carcinomas of the oesophagus. *Surg Gynecol Obstet* **177**: 299–302

Yoshida Y, Okamura T, Yano K, Taga S and Ezaki T (1994a) Histopathological characteristics associated with long term survival in stage III oesophageal carcinoma. *Cancer J* **7**(4): 147–149

Yoshida Y, Okamura T, Yano K and Ezaki T (1994b) Silver stained nucleolar organiser region proteins and *Helix pomatia* agglutinin immunostaining in oesophageal carcinoma – correlated prognostic factors. *J Surg Oncol* **56**(2): 116–121

Zak I, Lewandowska E and Gnyp W (2000) Review: Selectin glycoprotein ligands. *Acta Biochimica Polonica* **47**: 393–412

*© 2001 Cancer Research Campaign*
